# Identification of *PGC* as a Potential Biomarker for Progression from Barrett’s Esophagus to Esophageal Adenocarcinoma: A Comprehensive Bioinformatic Analysis

**DOI:** 10.3390/diagnostics14242863

**Published:** 2024-12-19

**Authors:** Sajida Qureshi, Waqas Ahmad Abbasi, Muhammad Asif Qureshi, Hira Abdul Jalil, Muhammad Saeed Quraishy

**Affiliations:** Dow Medical College, Dow University of Health Sciences, Karachi 74200, Pakistan; waqas.abbasi@duhs.edu.pk (W.A.A.); a.qureshi@duhs.edu.pk (M.A.Q.); dr.hkhan9@gmail.com (H.A.J.); saeed.quraishy@duhs.edu.pk (M.S.Q.)

**Keywords:** Barrett esophagus, esophageal adenocarcinoma, *PGC*, hub genes, bioinformatics, biomarker

## Abstract

**Background**: Barrett’s esophagus (BE), with metaplastic columnar epithelium in the lower esophagus, predisposes patients to esophageal adenocarcinoma (EAC). Despite extensive research, mechanisms underlying BE progression to EAC remain unclear, and no validated biomarkers are available for clinical use. Progastricsin/Pepsinogen-C (PGC), an aspartic proteinase linked to maintaining normal epithelial morphology, is often absent in advanced gastrointestinal malignancies. This study comprehensively investigates PGC expression across cancers, particularly in esophageal cancer (ESCA), to clarify its role in BE progression to EAC. **Methods**: We utilized multiple bioinformatic platforms (TIMER, UALCAN, cBioPortal, GEPIA, STRING, Metascape, and GEO database) to assess *PGC* expression, genomic alterations, and correlations with clinicopathological features, survival, and immune infiltration. Additionally, using the GEO dataset, we compared non-dysplastic Barrett’s esophagus (NDBE) patients with those who progressed to malignancy, identifying differentially expressed genes (DEGs), their interactions, and potential roles in progression. **Results**: *PGC* was notably upregulated in various cancers, especially in adjacent normal tissues of ESCA. Genomic amplifications of *PGC* were linked to improved survival in EAC patients, particularly those with high *PGC* expression, suggesting a protective role. Moreover, *PGC* expression positively correlated with favorable immune infiltration, notably B cells and CD8+ T cells. Enrichment analysis of downregulated DEGs revealed significant involvement in key biological processes, specifically in extracellular matrix organization. Among the downregulated DEGs, we identified *PGC* among the top 10 hub genes, underscoring its role in tissue homeostasis. **Conclusions**: These findings suggest that *PGC* could serve as a promising biomarker for predicting the high-risk transformation from BE to EAC, offering new insights into EAC progression and future therapeutic targets.

## 1. Introduction

Esophageal carcinoma (ESCA) is one of the most common cancers and ranks as the eighth leading cause of cancer-related deaths worldwide [1]. The majority of ESCAs are either esophageal squamous cell carcinomas (ESCC) or esophageal adenocarcinomas (EAC), originating from the esophageal epithelium. The etiology of ESCA remains poorly understood, and early-stage ESCAs typically at times are presented without any specific clinical symptoms. This often results in many patients missing the opportunity for early diagnosis. Consequently, the British and American Societies of Gastroenterology have developed comprehensive guidelines for the screening and surveillance of Barrett’s esophagus (BE) along with EAC [2,3,4,5].

BE, a condition in which the esophageal lining is replaced by tissue similar to the intestinal lining, is a well-established precancerous state of EAC, with poor survival outcomes [6]. Several well-established risk factors for BE include a history of smoking [7,8], chronic gastroesophageal reflux disease (GERD) [7,9], obesity [7,10], or a family history of BE or EAC [11]. Various methods have been developed for BE screening to detect the disease at the metaplastic state before progressing to dysplasia, such as endoscopy and cytosponge techniques [12].

Recent analyses indicate that the yearly transition rates from nondysplastic Barrett’s esophagus (NDBE) to EAC range between 0.33% and 0.70% [13,14,15,16,17,18]. As a result, endoscopic surveillance of NDBE is recommended every 3–5 years [4,19]. To date, no biomarkers have been validated for clinical use. Several molecular markers have been identified as useful in pinpointing patients with an increased risk of malignancy. Among the most extensively studied alterations in NDBE are those involving the tumor suppressor genes p53 and p16 [20]. However, there is a need for more robust markers, along with the ones currently being studied, to better understand the other factors contributing to the progression of BE towards malignant EAC.

The Progastricsin/Pepsinogen C (*PGC*) gene produces an aspartic proteinase belonging to the peptidase family A1. This enzyme is typically found in the normal stomach as a significant component of the gastric mucosa; being considered a negative marker in gastric cancer (GC), it is also synthesized in BE. Interestingly, PGC expression varies across cancers and is frequently upregulated in certain sex-related cancers, such as prostate, breast, and ovarian cancers, where higher levels are associated with improved prognosis [21,22,23,24]. Moreover, loss of pepsinogen in advanced ESCA, similar to GC, has been observed, suggesting some role in carcinogenesis [25,26,27].

Therefore, we aim to thoroughly investigate the expression profile of *PGC* with a pan-cancer approach, specifically in ESCA, and understand its significance in the progression of BE to EAC.

## 2. Materials and Methods

### 2.1. Pan-Cancer Expression Analysis and Validation

The Tumor Immune Estimation Resource (TIMER) (http://timer.cistrome.org/; accessed on 10 May 2024) database incorporates statistics from The Cancer Genome Atlas (TCGA), providing insights into expression patterns of various tumor and normal tissues. The “Gene DE” tool was employed to visually represent the pan-cancer distribution of PGC expression levels via box plots, and the significance was assessed using the Wilcoxon test.

Moreover, the University of Alabama at Birmingham Cancer Data Analysis Portal (UALCAN) facilitates expression analysis using TCGA datasets across 24 different cancers and their adjacent normal tissues. PGC expression was further validated through the “Pan-cancer view” component of UALCAN (https://ualcan.path.uab.edu/; accessed on 10 May 2024).

### 2.2. Genomic Alteration Analysis

Genomic alterations of PGC were assessed using the cBioPortal database (https://www.cbioportal.org/; accessed on 11 May 2024). Data from all 32 TCGA PanCancer Atlas studies, comprising 10,967 samples, were selected for analysis. The OncoPrint tool was employed to visualize overall alterations, with the data type set to “event per patient” to avoid duplication. The “Cancer Type Summary” feature was then used to visualize individual alterations among TCGA studies. Furthermore, for a more focused analysis, esophageal adenocarcinoma (EAC) data from the TCGA PanCancer Atlas, consisting of 182 samples, were specifically examined. The EAC TCGA dataset was downloaded and imported into SPSS version 24. The patient data were categorized into two groups according to amplification. Kaplan–Meier survival curves were plotted using overall survival status (alive or dead) and survival time (in months). The log-rank test was conducted to assess survival differences between the groups.

### 2.3. Correlation with Clinicopathological Features

The relationship between *PGC* mRNA expression levels and clinicopathological characteristics in ESCA was assessed using the UALCAN database (https://ualcan.path.uab.edu/; accessed on 12 May 2024). The analysis was conducted via the “Expression” feature and compared *PGC* expression against characteristics such as age, cancer stage, gender, tumor grade, tumor histology, and TP53 mutation status.

### 2.4. Overall Survival Analysis

To assess the influence of PGC expression on overall survival (OS), we utilized the Gene Expression Profiling Interactive Analysis (GEPIA) database (http://gepia.cancer-pku.cn/; accessed on 12 May 2024) to plot survival curves for 32 TCGA cancer types. GEPIA is a publicly accessible repository that includes RNA expression data from 9736 tumors and 8587 normal samples. Due to low sample sizes, some TCGA cancer types were excluded by (d default from the OS analysis.

Kaplan–Meier curves were employed to compare two patient groups (high and low PGC expression). Hazard ratios (HR), along with their corresponding 95% confidence intervals and log-rank *p*-values, were calculated. Additionally, the prognostic value of PGC expression in ESCA was validated using the UALCAN database (https://ualcan.path.uab.edu/; accessed on 12 May 2024).

### 2.5. Microarray Data

The Gene Expression Omnibus (GEO) database (http://www.ncbi.nlm.nih.gov/geo; accessed on 1 June 2024) is a publicly accessible repository for genomic data. For this study, we selected the gene expression profile dataset GSE210647, which is based on the Illumina NextSeq 500 GPL18573 platform and includes 29 samples. These samples encompass nondysplastic Barrett’s esophagus (NDBE) tissues, BE samples that progressed to malignancy, and normal tissues as controls. The selection of this dataset was based on the following criteria: (a) inclusion of two or more groups of tissues to enable comparative analysis, with at least one group representing progression to esophageal malignancy; (b) a sample size greater than 10; and (c) data updated within the last two years (2022–2024).

### 2.6. Data Processing of DEGs

Differentially expressed genes (DEGs) between Barrett’s esophagus and Barrett’s esophagus samples that progressed to malignancy were identified using GEO2R (https://www.ncbi.nlm.nih.gov/geo/geo2r/; accessed on 1 June 2024), an interactive online tool designed for comparing sample groups within a GEO Series. To minimize false positives, we utilized adjusted *p*-values, employing the Benjamini and Hochberg method as default. The cut-off criteria for identifying upregulated and downregulated DEGs were set at an adjusted *p*-value < 0.05 and |logFC| ≥ 3.5

### 2.7. Protein–Protein Interaction Network and Enrichment Analysis

To elucidate the direct and indirect interactions among the downregulated DEGs, the Search Tool for the Retrieval of Interacting Genes/Proteins (STRING) (http://string-db.org; accessed on 1 June 2024) was used to construct a protein–protein interaction (PPI) network. We set a minimum interaction score of “medium confidence” (0.400) and considered network edges based on various evidence sources, such as Textmining, Experiments, Databases, Coexpression, Neighborhood, Gene Fusion, and Co-occurrence.

Subsequently, enrichment analysis was performed using Metascape (https://metascape.org/gp/index.html#/main/step1; accessed on 3 June 2024), which is an online platform for gene functional enrichment analysis. Metascape provides a visual representation of the results by analyzing the gene/protein lists. We used the “Custom Analysis” mode of Metascape to conduct Gene Ontology (GO) analysis, including Biological Process (BP), Cellular Component (CC), and Molecular Function (MF), as well as KEGG pathway analysis. A *p*-value of <0.01 was considered significant, with a minimum enrichment value of 1.5.

### 2.8. Hub Gene Screening

Cytoscape software (version 3.10.1) was employed to identify the top 10 hub genes within the network. Using the CytoHubba plugin in Cytoscape, we used the degree method to determine these hub genes, highlighting the shortest paths and key nodes within the network.

### 2.9. Validation of Hub Genes

We utilized the GEPIA tool to confirm the expression levels of the identified hub genes (http://gepia.cancer-pku.cn/; accessed on 7 June 2024). The genomic expression levels of the top 10 hub genes were assessed in ESCA using TCGA tumor and normal tissue data.

### 2.10. Immune Infiltration Analysis

To investigate the correlation between *PGC* expression and immune infiltration levels in ESCA, the TIMER tool (https://cistrome.shinyapps.io/timer/; accessed on 8 June 2024) was utilized. The gene module function was employed to analyze the correlation of *PGC* expression within ESCA, focusing on infiltrates such as B cells, CD8+ T cells, CD4+ T cells, macrophages, neutrophils, and dendritic cells. Correlations were visualized through scatter plots, and statistical significance was assessed using Spearman’s correlation coefficient. *p* values ≤ 0.05 were considered statistically significant.

## 3. Results

### 3.1. Upregulation of PGC in Various TCGA Tumors and ESCA-Adjacent Normal Tissue

To examine the expression of PGC in a pan-cancer context, we analyzed its expression levels in various tumors and corresponding normal tissues using the TIMER database. Our inquiry discovered significant upregulation of PGC expression in numerous cancers, including colon adenocarcinoma (COAD), rectum adenocarcinoma (READ), kidney renal clear cell carcinoma (KIRC), kidney renal papillary cell carcinoma (KIRP), glioblastoma (GBM), liver hepatocellular carcinoma (LIHC), lung adenocarcinoma (LUAD), lung squamous cell carcinoma (LUSC), skin cutaneous melanoma (SKCM), cholangiocarcinoma (CHOL), and uterine corpus endometrial carcinoma (UCEC), compared to their normal counterparts. While PGC expression was also notable in breast carcinoma (BRCA), prostate adenocarcinoma (PRAD), and stomach adenocarcinoma (STAD), these did not reach statistical significance. The remaining cancer types showed either minimal or comparable expression to adjacent normal tissues.

**Figure 1:**
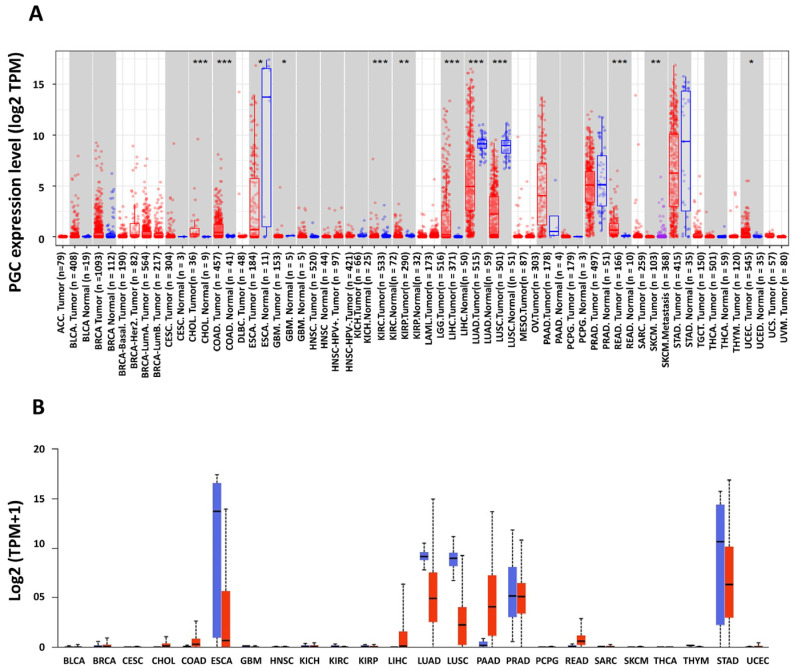
Differential expression of PGC in various cancers and adjacent normal tissues. (**A**) mRNA expression levels of PGC across TCGA cancers and their normal tissues using the TIMER-2 database. Statistical significance is indicated by asterisks: * *p* < 0.05, ** *p* < 0.01, and *** *p* < 0.001. (**B**) Validation of PGC expression analysis between pan-cancers and corresponding normal tissues, analyzed using the UALCAN database. Expression levels are quantified in transcripts per million (TPM) and presented on a logarithmic scale.

Interestingly, a significant upregulation of PGC was observed in adjacent normal tissues to ESCA (Figure 1A). These findings were marked by asterisks (*: *p*-value < 0.05; **: *p*-value < 0.01; ***: *p*-value < 0.001). To validate this finding, we further utilized the UALCAN database, which further confirmed the prominent expression of PGC in adjacent normal ESCA tissues (Figure 1B).

### 3.2. PGC Genomic Alterations in Esophageal Adenocarcinoma

**Figure 2:**
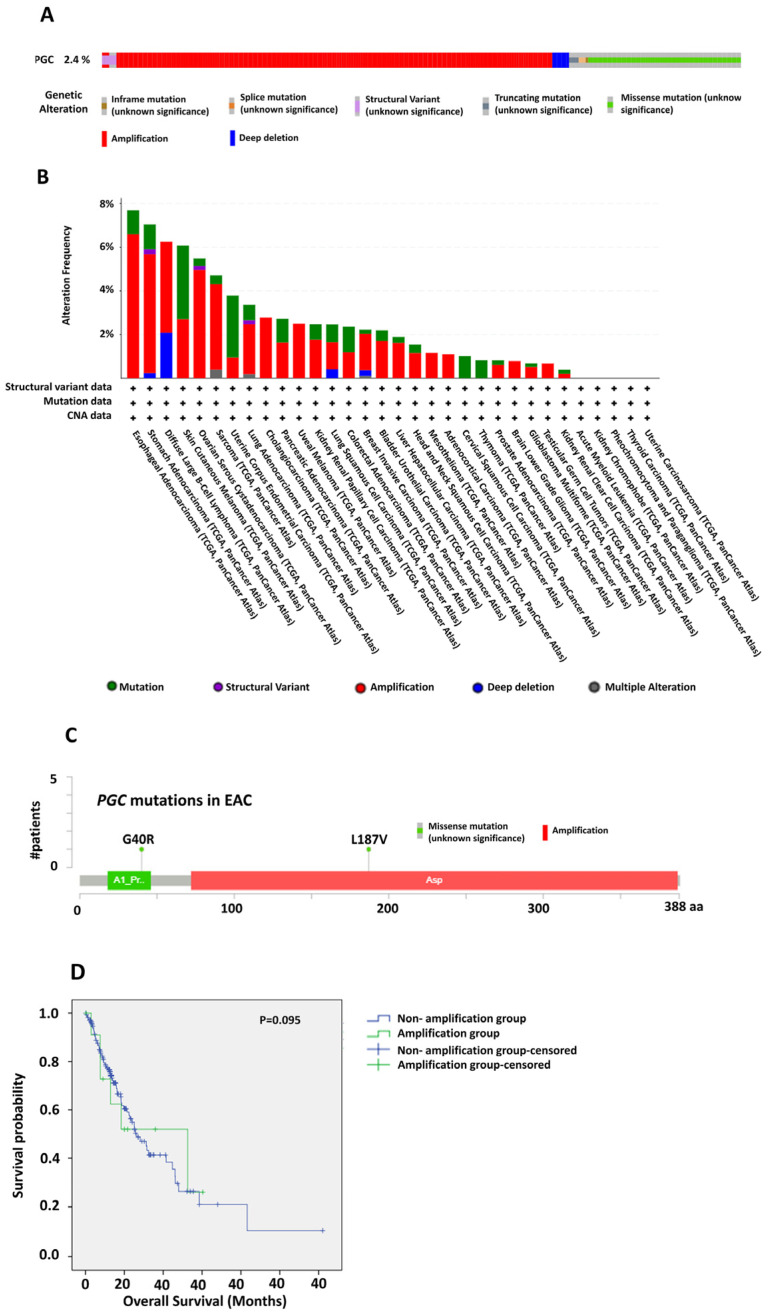
Genetic alterations of PGC among TCGA cancers. (**A**) Assessment of overall genetic alterations of PGC across 32 TCGA PanCancer Atlas studies using the cBioPortal database. (**B**) Frequency distribution of PGC genetic alterations among individual TCGA studies. (**C**) Specific analysis of PGC mutations in ESCA, including amplification and missense mutations along the amino acid sequence. (**D**) Overall survival analysis between patients with PGC amplification and those without.

We investigated the genetic alterations of *PGC* across 32 TCGA PanCancer Atlas studies using the cBioPortal database, encompassing 10,953 patients. The analysis indicated that *PGC* is genetically altered in 2.4% (*n* = 268) of the samples. These alterations included amplifications, deep deletions, in-frame mutation, splice mutation, structural variant, truncating mutation, and missense mutation, all of unknown significance (Figure 2A). The frequency distribution and type of these alterations varied among different cancer types (TCGA Atlas studies), with most alterations observed in the esophageal adenocarcinoma TCGA dataset (7.6%), predominantly involving amplifications along with missense mutations (Figure 2B).

Specifically, in the EAC dataset, missense mutations included the substitution of glycine (G) at position 40 to arginine (R) and leucine (L) at position 187 to valine (V), along with several amplification events along the *PGC* amino acid sequence (Figure 2C). Furthermore, OS was plotted between the amplification and nonamplification groups with the same EAC dataset. Although statistical significance was not achieved (*p* = 0.095), the analysis depicted that patients with *PGC* amplification had better overall survival in comparison to the other group, highlighting the importance of *PGC* expression complemented by amplification in EAC (Figure 2D).

### 3.3. Correlation of PGC Expression with Clinicopathological Characteristics

**Figure 3:**
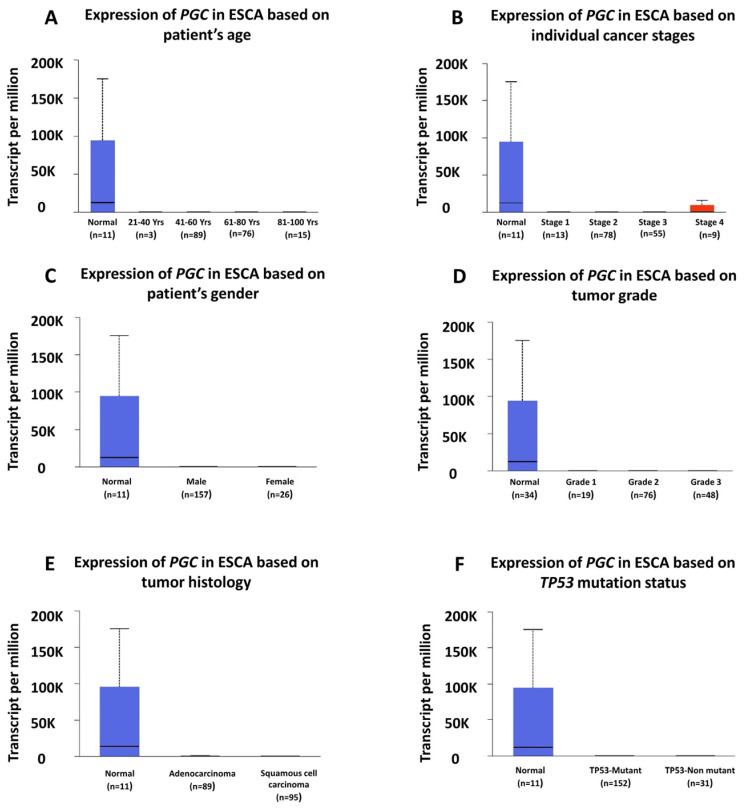
Clinicopathological features associated with PGC expression in ESCA and adjacent normal tissues. The UALCAN database was used, and PGC expression was analyzed based on (**A**) patient age, (**B**) cancer stage, (**C**) gender, (**D**) tumor grade, (**E**) tumor histology, and (**F**) TP53 mutation status.

We investigated the relationship between *PGC* expression and various clinicopathological characteristics in ESCA and adjacent normal tissues, including the patient’s age (Figure 3A), cancer stages (Figure 3B), patient gender (Figure 3C), tumor grade (Figure 3D), tumor histology (Figure 3E), and TP53 mutation status (Figure 3F).

Our findings showed that *PGC* expression was considerably higher in normal tissues across all age groups, gender, cancer stages, and tumor grades in ESCA, indicating its independence from these variables. *PGC* was also more prominently expressed in normal tissues compared to different histological types (ESCC and EAC) of ESCA, as well as maintaining its pattern regardless of TP53 mutation status.

### 3.4. Prognostic Significance of PGC Expression

Survival analysis was performed using the GEPIA tool to evaluate the prognostic value of *PGC* expression across various cancers. High *PGC* expression was linked to poor OS in several cancer types, including KIRC (log-rank *p*-value = 0.029) and MESO (log-rank *p*-value = 0.029). Conversely, some cancer types showed poor overall survival in the low *PGC* expression group compared to the high expression group. Notably, among them, GBM (log-rank *p*-value = 0.021) showed a significant difference.

**Figure 4:**
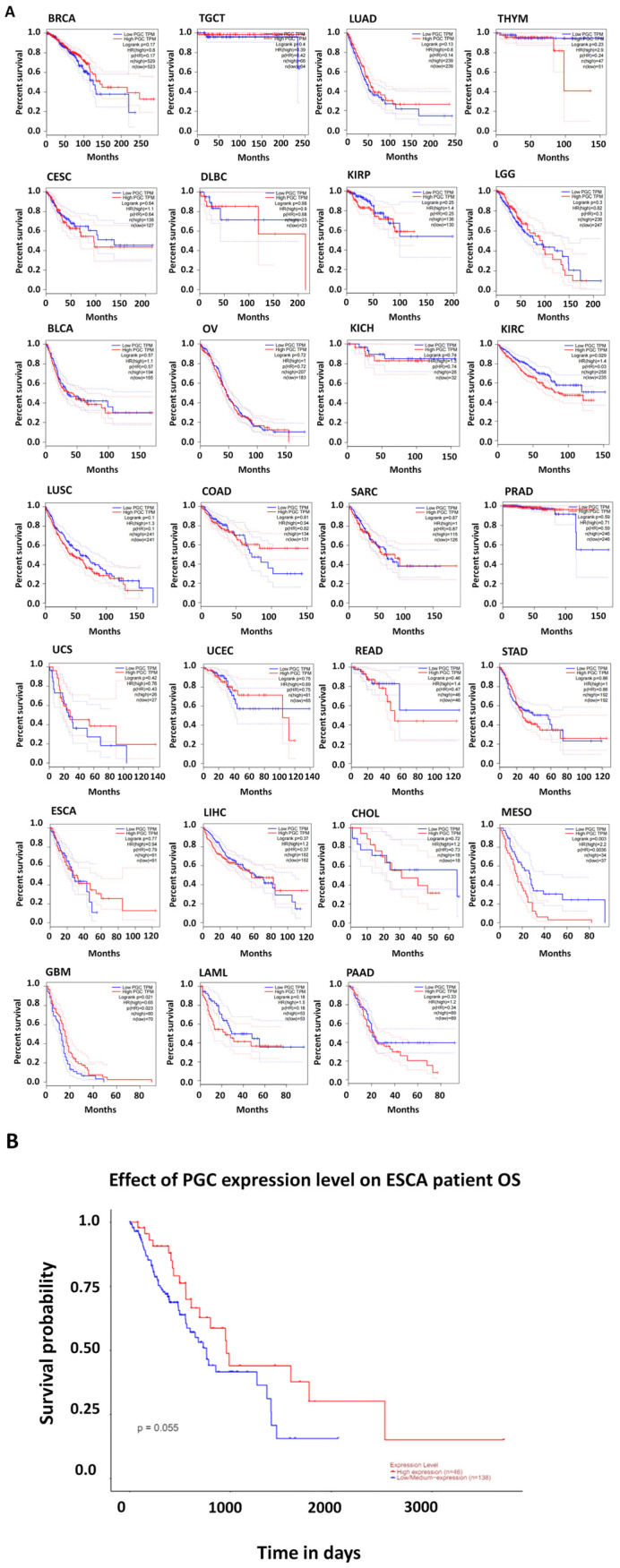
Prognostic value of PGC in cancers. (**A**) OS analysis comparing high and low PGC expression patient groups across TCGA cancers performed using the GEPIA 2 web tool. (**B**) Validation of PGC expression’s impact on OS specifically in ESCA patients via UALCAN database.

Additionally, other cancer types such as BRCA, LUAD, KIRP, PRAD, UCS, STAD, LIHC, TGCT, and specifically ESCA demonstrated worse survival outcomes in the low *PGC* expression groups. Although these cancer types showed a trend of decreased survival probability in low *PGC* expression patients, statistical significance was not achieved (Figure 4A).

We validated the low expression and unfavorable prognostic value of *PGC* in ESCA using the UALCAN database. This analysis revealed significant findings indicating a correlation between low *PGC* expression and poor OS in ESCA patients (*p*-value = 0.055) (Figure 4B). These results propose that *PGC* could serve as an important prognostic marker in ESCA, as well as potentially in other cancers where low *PGC* expression is associated with poorer outcomes.

### 3.5. Role of PGC in the Malignant Progression of Barrett’s Esophagus

To explore and validate the significance of *PGC* in ESCA, we analyzed a dataset from the GEO database (Accession Number: GSE210647, *n* = 29). This dataset consisted of high-throughput sequencing data from patients with NDBE, including patients who later progressed to high-grade dysplasia (HGD) or EAC, as well as some of those who did not progress towards malignancy. We defined the groups based on progression and executed GEO2R analysis to understand *PGC*’s potential role and identify other partners contributing to the malignant progression of NDBE.

**Figure 5:**
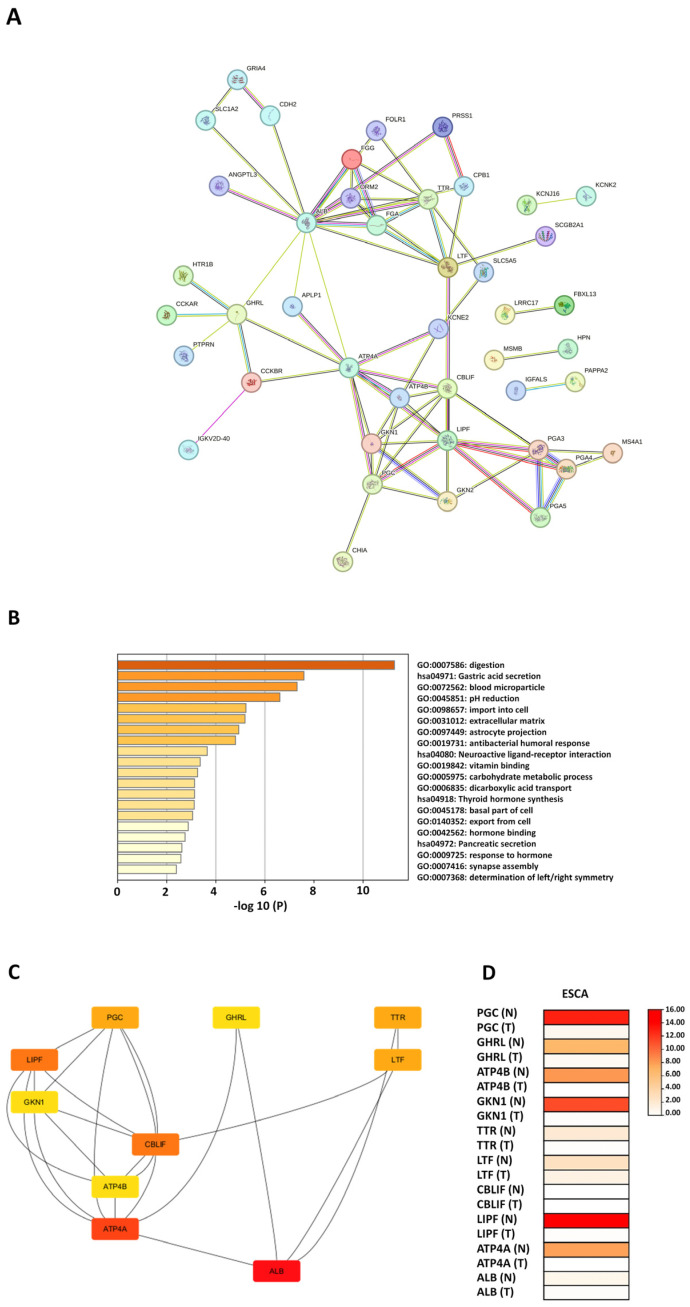
Construction of the PPI network, functional enrichment analysis, and screening of hub genes for downregulated DEGs. (**A**) PPI network constructed for the downregulated DEGs using the STRING tool. (**B**) Functional enrichment analysis of the downregulated DEGs performed using Metascape. (**C**) Top 10 hub genes identified using the CytoHubba plugin in Cytoscape. (**D**) Validation of top 10 hub gene expression using the GEPIA tool.

We identified a total of 227 DEGs, consisting of 152 upregulated and 75 downregulated genes (Table 1). Subsequently, we set up the PPI network for the downregulated DEGs via the STRING tool, with a confidence level > 0.4. This analysis revealed significant interactions among the downregulated genes, highlighting key nodes and edges within the network (number of nodes = 65, number of edges = 77, average node degree = 2.37, average local clustering coefficient = 0.472, expected number of edges = 11, and PPI enrichment *p*-value < 1.0 × 10^−16^) (Figure 5A).

Following PPI plotting, we performed enrichment analysis on the 75 downregulated genes using Metascape, which revealed involvement in several significant biological processes and pathways (Figure 5B). The key terms identified included digestion (GO:0007586), gastric acid secretion (hsa04971), blood microparticle (GO:0072562), pH reduction (GO:0045851), import into cell (GO:0098657), extracellular matrix (ECM) (GO:0031012), astrocyte projection (GO:0097449), antibacterial humoral response (GO:0019731), neuroactive ligand–receptor interaction (hsa04080), vitamin binding (GO:0019842), carbohydrate metabolic process (GO:0005975), dicarboxylic acid transport (GO:0006835), thyroid hormone synthesis (hsa04918), basal part of cell (GO:0045178), export from cell (GO:0140352), hormone binding (GO:0042562), pancreatic secretion (hsa04972), response to hormone (GO:0009725), synapse assembly (GO:0007416), and determination of left/right symmetry (GO:0007368). Some of these enriched terms highlight the unique biological roles of the downregulated genes and their potential contributions to the malignant progression of EAC.

The PPI network was then imported into Cytoscape, where we utilized the CytoHubba plugin to identify the top 10 hub genes (*ALB*, *ATP4A*, *LIPF*, *CBLIF*, *LTF*, *TTR*, *PGC*, *GKN1*, *ATP4A*, and *GHRL*) based on the degree method (Figure 5C). Among the hub genes identified, all except *CBLIF* showed higher expression levels in normal tissues compared to tumor tissues in ESCA. Specifically, *PGC*, *LIPF*, and *GKN1* exhibited the most significant upregulation in normal tissues, followed by *GHRL* and *ATP4B* (Figure 5D).

### 3.6. Correlation of PGC Expression with Immune Infiltration

We investigated the relationship between *PGC* expression and levels of immune infiltration in ESCA using the TIMER tool. The analysis displayed the gene expression level against tumor purity in the leftmost panel, revealing a negative association and signifying low tumor purity in the microenvironment.

**Figure 6:**
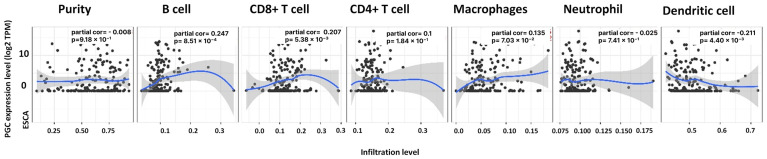
Relationship between PGC expression and immune infiltration in ESCA. Scatter plots showing the correlation between PGC expression and infiltration levels of various immune cell types (B cells, CD8+ T cells, CD4+ T cells, macrophages, neutrophils, and dendritic cells) in ESCA, with statistical significance assessed using Spearman’s correlation coefficient.

Our findings revealed significant correlations between *PGC* expression and several immune cell types. Specifically, *PGC* expression showed a significant positive correlation with B cells (partial correlation = 0.247, *p* = 8.51 × 10^−4^) and CD8+ T cells (partial correlation = 0.207, *p* = 5.38 × 10^−3^). Moreover, the correlations between *PGC* expression and macrophages, CD4+ T cells, and neutrophils were positive but not statistically significant. Conversely, a negative correlation was observed with dendritic cells (partial correlation = −0.211, *p* = 4.40 × 10^−3^) (Figure 6).

## 4. Discussion

In this study, we identified *PGC* as a potentially significant marker, with its expression patterns and genomic alterations indicating prognostic value, implications for immune modulation, and notable enrichment in key biological processes.

EAC has a poor prognosis [28], while BE increases the risk of developing EAC fourfold [2]. Efforts to identify early detection biomarkers for BE are ongoing, with numerous trials in progress [29], but there remains a gap in identifying and validating molecular markers that predict progression from BE to EAC. Tan et al. [30] observed that the incidence rates of EAC and HGD/EAC remain relatively low for NDBE. In contrast, our in silico findings identified significant factors potentially contributing to the progression from NDBE to EAC, emphasizing the utility of markers such as *PGC* in risk stratification. To our knowledge, this is the first comprehensive in silico analysis where *PGC* has been investigated for its significance in BE progression to EAC.

Among the promising biomarkers, altered *TP53* tissue expression has been highlighted for risk stratification. Despite its potential, *TP53* has shown variable clinical utility, with only 49% of patients who progressed to EAC demonstrating aberrant *TP53* immunostaining [29]. Additionally, a nested case–control study found that *TP53* protein overexpression did not reliably predict progression to EAC [31], which limits its clinical utility.

Our study includes a pan-cancer expression analysis of *PGC* across all TCGA cancers, revealing its broad-spectrum presence and suggesting that *PGC* could serve as a diverse biomarker/target in human cancers. Moreover, our findings propose an interesting potential tumor-suppressive/control role for *PGC* in ESCA, possibly due to its involvement in complex pathways [25]. Gastrointestinal malignancies exhibit some shared molecular events across their genetic landscapes [27]. In GC, *PGC* expression is crucial for preserving normal epithelial cell morphology and function; however, expression significantly decreases during the progression to advanced GC stages [32]. Similarly, our results demonstrate significant expression of *PGC* in adjacent normal tissues to ESCA, with minimal expression in ESCA tumors. Shen et al. [25] confirm that *PGC* was not detected at the protein level in ESCA tissues, validating its role in upholding normal tissue homeostasis.

Moreover, *PGC*’s function as a zymogen is still somewhat unclear [33], which contrasts with the function of pepsin, which is generally linked to tumorigenesis in ESCA [27,34]. This highlights the complex role of the pepsinogen family in ESCA as well as in normal tissue biology [25]. The prognostic value of some pepsinogen family members, particularly *PGC*, has been established for other cancers [33]. Notably, our findings reveal that *PGC* amplifications, leading to high expression levels, are associated with better survival in ESCA patients. This reinforces the protective role of *PGC* in maintaining tissue homeostasis, as well as underscoring its prognostic significance.

Additionally, in our analysis of the DEGs, the enrichment analysis of the downregulated genes provides valuable insights, highlighting involvement in processes such as digestion, pH reduction, gastric acid secretion, and ECM organization, among others. Changes in ECM stiffness can trigger mechanotransduction signaling, regulating malignant behaviors and activating oncogenic pathways [35,36]. These findings underscore the potential of some downregulated genes as biomarkers and strategic therapeutic targets.

We identified the top 10 hub genes through the downregulated PPI network: *ALB, ATP4A, LIPF, CBLIF, LTF, TTR, PGC, GKN1, ATP4A,* and *GHRL*. While our primary focus was on *PGC*, our results also highlight a range of other key genes, suggesting a potentially cohesive involvement in the high-risk transformation from BE to EAC. Recent studies using a similar methodology strategy, with both generalized and specific approaches, have aimed to identify important networks and hub genes associated with BE progression to EAC [37,38,39]. These studies reported various significant hub genes, different from ours, likely due to the diversity of the GEO datasets and studied cohorts. Our findings, therefore, contribute uniquely to the understanding of the molecular mechanisms underlying progression to EAC, adding to the available network data and further emphasizing the importance of context-specific analyses.

Furthermore, the role of immune cell infiltration in ESCA has gained attention for its potential prognostic and therapeutic implications. *PGC* is notable for its strong correlation, showing promise as a potential immune-related biomarker in BE for modulating immune responses [25,40]. Our findings align with and further extend these concepts, validating the significant infiltration of B cells and CD8+ T cells, highlighting a protective immune microenvironment in ESCA.

However, it is essential to acknowledge the potential limitations inherent in bioinformatics analyses. These limitations may arise from the reliance on publicly available datasets, which may not adequately capture the complexities of tumor biology, coupled with the lack of experimental validation to substantiate our findings. Future research should include in vitro validations to confirm these relationships and establish definitive cause-and-effect linkages.

In conclusion, our comprehensive analysis underscores the significance of *PGC* as a potential biomarker for high-risk transformation from BE to EAC. Our findings enhance the understanding of the molecular mechanisms driving progression to EAC and provide important leads for novel therapeutic approaches and prognostic assessments in the future.

## Figures and Tables

**Table 1:** DEGs extracted from GEO series (GSE210647).

DEGs	Gene Symbols
Upregulated genes (152)	*ESPN, SLC2A5, RNF186, PLA2G2A, HTR1D, GUCA2B, GUCA2A, BEST4, SLC44A5, CLCA1, CLCA3P, PDZK1, PDZK1P1, KPRP, SPRR2F, AQP10, RHBG, FMO2, SLC30A10, LOC105373475, LEFTY1, LEFTY2, APOB, KHK, SLC8A1-AS1, ABCG5, ABCG8, NAT8B, FABP1, MYO7B, TM4SF20, ALPI, AGXT, NEU4, SSUH2, C3orf85, TM4SF4, SI, TPRG1, SLC51A, GBA3, LOC101929161, SLC34A2, UGT2B7, ADH4, MTTP, FABP2, F11, SLC6A19, LOC340090, NPY6R, SLC17A4, HCG22, MLN, LRFN2, MEP1A, TINAG, LINC02535, VNN1, UNC93A, NPC1L1, CLDN3, CYP3A4, MUC12, MOGAT3, CLDN15, SLC26A3, LOC105375469, DEFA6, DEFA5, NAT2, CA1, CDH17, LOC105375647, FAM83A, FAM83A-AS1, ANXA13, LYNX1-SLURP2, LRRC19, ALDOB, C8G, TUBAL3, TMEM236, MALRD1, DKK1, PLA2G12B, MYOZ1, ABCC2, PRAP1, CDHR5, TBX10, HEPHL1, APOA4, APOC3, APOA1, APOA1-AS, KRT72, SLC39A5, LINC02404, HAL, SLC17A8, LINC02826, ATP12A, CDX2, LINC00330, ZIC5, ZIC2, TMEM253, FLVCR2-AS1, FLVCR2, DIO2-AS1, SLC51B, ANPEP, CASP16P, CHP2, CA7, CHST5, SLC52A1, ALOX12, NOS2, C17orf78, KRT24, KRT31, FADS6, OTOP3, ANKRD62, CYP4F35P, MEP1B, REEP6, CREB3L3, CCL25, CYP4F2, SLC7A9, CYP2B6, ERICH4, TEX101, CEACAM20, LOC105372442, KLK12, CEACAM18, WFDC12, PCK1, EDN3, TMPRSS15, PRODH, LINC02891, LOC102724788, ARSF, ACE2, OTC, XPNPEP2, ADGRG4.*
Downregulated genes (75)	*KANK4, ANGPTL3, CHIA, PAPPA2, MFSD4A, SLC26A9, KCNK2, LINC02934, GKN2, GKN1, IGKV2D-40, CFC1B, CFC1, PTPRN, DNER, GHRL, LTF, CPB1, PSAPL1, CCKAR, ALB, ETNPPL, NDST3, FGA, FGG, SPINK13, PGC, HTR1B, FUT9, TRIM50, FBXL13, LRRC17, PRSS1, SH3GL2, ORM2, MSMB, LIPF, CCKBR, SLC1A2, CBLIF, MS4A1, PGA3, PGA4, PGA5, SCGB2A1, TMEM151A, SMIM38, FOLR1, GRIA4, B3GAT1, ATP4B, SYT16, ELK2AP, IGHG1, IGHG3, IGHV4-30-2, IGHV1-58, IGFALS, C16orf89, KCNJ16, AQP4, CDH2, TTR, SLC5A5, HPN, ATP4A, APLP1, CKM, SIGLEC11, ZSCAN4, LOC105372594, LOC105372791, KCNE2, IGLV1-41, MAP7D2.*

DEG, differentially expressed gene; GEO, Gene Expression Omnibus.

## Data Availability

All data used in this study are publicly available, with sourcing details provided in the methodology section. Additionally, data can be obtained from the corresponding author upon request.
